# Early Combination Antiretroviral Therapy Limits Exposure to HIV-1 Replication and Cell-Associated HIV-1 DNA Levels in Infants

**DOI:** 10.1371/journal.pone.0154391

**Published:** 2016-04-22

**Authors:** Margaret McManus, Eric Mick, Richard Hudson, Lynne M. Mofenson, John L. Sullivan, Mohan Somasundaran, Katherine Luzuriaga, PACTG 356 Investigators

**Affiliations:** 1 Program in Molecular Medicine, University of Massachusetts Medical School, Worcester, Massachusetts, United States of America; 2 Department of Quantitative Health Sciences, University of Massachusetts Medical School, Worcester, Massachusetts, United States of America; 3 The Elizabeth Glaser Pediatric AIDS Foundation, Washington DC, United States of America; University of Pittsburgh, UNITED STATES

## Abstract

The primary aim of this study was to measure HIV-1 persistence following combination antiretroviral therapy (cART) in infants and children. Peripheral blood mononuclear cell (PBMC) HIV-1 DNA was quantified prior to and after 1 year of cART in 30 children, stratified by time of initiation (early, age <3 months, ET; late, age >3 months-2 years, LT). Pre-therapy PBMC HIV-1 DNA levels correlated with pre-therapy plasma HIV-1 levels (r = 0.59, p<0.001), remaining statistically significant (p = 0.002) after adjustment for prior perinatal antiretroviral exposure and age at cART initiation. PBMC HIV-1 DNA declined significantly after 1 year of cART (Overall: -0.91±0.08 log_10_ copies per million PBMC, p<0.001; ET: -1.04±0.11 log_10_ DNA copies per million PBMC, p<0.001; LT: -0.74 ±0.13 log_10_ DNA copies per million PBMC, p<0.001) but rates of decline did not differ significantly between ET and LT. HIV-1 replication exposure over the first 12 months of cART, estimated as area-under-the-curve (AUC) of circulating plasma HIV-1 RNA levels, was significantly associated with PBMC HIV-1 DNA at one year (r = 0.51, p = 0.004). In 21 children with sustained virologic suppression after 1 year of cART, PBMC HIV-1 DNA levels continued to decline between years 1 and 4 (slope -0.21 log_10_ DNA copies per million PBMC per year); decline slopes did not differ significantly between ET and LT. PBMC HIV-1 DNA levels at 1 year and 4 years of cART correlated with age at cART initiation (1 year: p = 0.04; 4 years: p = 0.03) and age at virologic control (1 and 4 years, p = 0.02). Altogether, these data indicate that reducing exposure to HIV-1 replication and younger age at cART initiation are associated with lower HIV-1 DNA levels at and after one year of age, supporting the concept that HIV-1 diagnosis and cART initiation in infants should occur as early as possible.

## Introduction

Control of HIV-1 replication following the initiation of combination antiretroviral therapy (cART) in the first few months following birth preserves CD4+ T cell counts and general immune function and prevents HIV-1 associated disease progression in infants [1, 2]. Early combination antiretroviral therapy can also markedly reduce HIV-1 associated mortality [3]. Current guidelines [4, 5] thus recommend early infant diagnosis and the immediate initiation of cART in all HIV-1 infected infants under 12 months of age.

While cART may control HIV-1 replication to the point that plasma HIV-1 RNA levels are undetectable by routine and ultrasensitive assays, HIV-1 DNA remains detectable in circulating CD4+ T cells. The observation that most children, including those with stable, long-term suppression of HIV-1 replication on cART, experience a rebound in viral replication within weeks of discontinuing therapy [6, 7] is compatible with the notion that at least some of the detectable cell-associated HIV-1 DNA is replication-competent; long-lived memory CD4+ T cells that harbor replication-competent HIV-1 (“latent reservoir”) serve as a barrier to cure [8, 9].

Low circulating levels of HIV-1 DNA and smaller latent reservoir size have been measured in adults who have persistently controlled HIV-1 replication off cART following treatment in primary infection [10, 11]. PBMC HIV-1 DNA levels can be readily measured using the small blood volumes available from infants while *in vitro* viral outgrowth assays that measure the fraction of replication-competent HIV-1 require relatively large blood volumes (Reviewed in [8]). Cross-sectional studies have demonstrated lower levels of circulating HIV-1 DNA in children who suppressed HIV-1 replication prior to one year of age than after one year of age [12–14]. However, data quantifying HIV-1 persistence in children before and immediately following early cART are limited. We undertook this study to quantify PBMC HIV-1 DNA levels before and up to four years following early cART in children, with the specific goal of examining the relationships between circulating PBMC HIV-1 DNA levels to the timing of cART initiation and the duration of viremic exposure over the first year of treatment.

## Materials and Methods

### Study Cohort

The study cohort included 30 HIV-1 infected children (Table 1), stratified by timing of cART initiation (early therapy, <3 months of age, ET; late therapy, >3 months to 2 years, LT), for whom sufficient cryopreserved PBMC were available to measure HIV-1 DNA prior to and after 1 year of cART. Twenty-eight children received cART through an open-label, Phase I/II clinical trial (Pediatric AIDS Clinical Trials Group Protocol, PACTG 356 (NCT00000872, [6]) and two were treated by open prescription. HIV-1 DNA levels were measured yearly thereafter up to 4 years following cART initiation in 21 children who achieved plasma HIV-1 RNA levels of <400 copies/ml by 48 weeks of therapy and who sustained plasma HIV-1 RNA < 50 copies/ml thereafter (“virologic responders”). Children were excluded from analyses when plasma HIV-1 RNA was again detected at levels >50 copies/ml. Institutional Review Boards at the University of Massachusetts Medical School and at the clinical sites participating in PACTG 356 approved this study. Signed informed consent was obtained from all study participants’ guardians.

**Table 1:** Study Cohort: Pre-therapy Viral Load, CD4%, and Therapeutic Regimens^a^.

Cohort	N	Age (mos)	log_10_ Plasma HIV-1 RNA (copies/ml)	log_10_ HIV-1 DNA (copies/million PBMCs)	CD4%	Therapeutic Regimens
**All subjects**	30	2.9 (1.6–6.5)	5.3 (4.9–5.6)	3.4 (2.8–3.9)	40 (26–47)	19 (2 NRTIs + 1 NNRTI + 1 PI)
						9 (3 NRTIs + 1 NNRTI)
						2 (2 NRTIs + 1 NNRTI)
**ET Responders** ^b^	13	1.9 (1.4–2.3)	5.3 (5.1–5.6)	3.0 (2.7–3.4)	36 (28–57)	10 (2 NRTIs + 1 NNRTI + 1 PI)
						2 (3 NRTIs + 1 NNRTI
						1 (2 NRTIs + 1 NNRTI)
**LT Responders** ^b^	8	9.6 (5.7–13.3)	4.8 (3.6–5.7)	3.7 (3.0–3.9)	38 (28–43)	5 (2 NRTIs + 1 NNRTI + 1 PI)
						2 (3 NRTIs + 1 NNRTI)
						1 (2 NRTIs + 1 NNRTI)

^a^ Values are provided as medians with 25^th^ and 75^th^ percentiles (IQR).

^b^ Responders were defined as study subjects who achieved a viral load of <400 copies/ml by 48 weeks of cART therapy and whose peripheral blood HIV-1 RNA levels remained under assay detection limits through 4 years of cART.

### Quantitation of circulating HIV-1 RNA and total HIV-1 DNA in PBMC

Blood collected in tubes containing either acid citrate dextrose or EDTA (Becton-Dickinson, Mountainview, CA) was processed to collect plasma and to prepare PBMC pellets using Ficoll-Paque (Pharmacia Fine Chemicals, Piscataway, NJ) density gradient separation [15]. Plasma HIV-1 RNA load was measured using either the Roche Standard (LOD <400 copies/ml) or UltraSensitive (LOD <50 copies/ml) AMPLICOR HIV-1 MONITOR kits Version 1.5 (Roche Diagnostics, Indianapolis, IN).

Following lysis of PBMC pellets, HIV-1 DNA copy numbers in the cell lysates were quantified using the Amplicor HIV-1 DNA PCR assay version 1.5 (Roche Diagnostics, Branchburg, NJ) in a limiting dilution assay (LDA) format with endpoint calculations using the “Quality” computer program, as previously described [15]. Lysates of HIV-1 uninfected PBMC spiked with standardized numbers of 8E5 T cells (each containing a single copy of HIV-1 DNA) were used to determine the lower limit of endpoint detection (PCR-positive reaction) of the assay (2 copies of HIV-1 DNA per PCR), which is comparable to the detection limits reported using droplet digital PCR and real-time qPCR [7, 10, 16]. DNA levels were expressed as copies per 10^6^ PBMC with a detection limit of ~3–5 copies per 10^6^ PBMC.

### Statistical Analyses

Analyses used the software Stata (v13, StataCorp.) and GraphPad Prism version 6.05 for Windows, (GraphPad Software, San Diego California USA, www.graphpad.com). Correlations for log-transformed data were calculated using Pearson (r); for non-transformed data, Spearman rho was calculated. Generalized linear models (logistic regression for binary outcomes and ordinary least squares regression or continuous data) were fit to test for association with response status or plasma HIV-1 RNA copy numbers. Testing for association between concurrent pre-therapy plasma HIV-1 RNA and PBMC HIV-1 DNA values used linear regression models correcting for potentially confounding covariates. We quantified the degree of exposure to viral replication over the first 12 months of therapy by calculating the area-under-the-curve (AUC) of the circulating plasma HIV-1 RNA levels using the trapezoidal rule. The AUC estimation was limited to 1 year after treatment initiation because sampling after one year was less frequent than prior to 1 year. Linear regression models were used to predict one-year HIV-1 DNA values from the HIV-1 RNA AUC calculated from the previous 12 months, adjusting for potentially confounding covariates. Repeated PBMC HIV-1 DNA levels collected longitudinally were tested with linear mixed-regression models to estimate relative decline rates in ET and LT treated groups. The primary test of statistical significance is the time by treatment group interaction, which tests for differences in the shape of treatment-specific response curves. Additional post-estimation contrasts were used to compare group-means between groups at specific time points, the rate of decline from year 0 to 1 between groups, and further declines from one year to year 4 using reverse adjacent contrasts. All statistical tests were two-sided and p<0.05 was considered statistically significant.

## Results

### Rapid decline in PBMC HIV-1 DNA levels over the first year of cART

Thirty children, aged 1 month to 19 months, stratified by age at initiation of therapy (< 3 months, ET or ≥ 3 months-2 years, LT) were included in the study. Overall, the median age of children at cART initiation was 2.9 months and the median pre-therapy plasma HIV-1 RNA level was 5.3 log_10_ copies/ml (IQR: 4.9 to 5.6 copies/ml). The median pre-therapy PBMC HIV-1 DNA level was 3.4 log_10_ copies/ million PBMC (IQR: 2.8–3.9 log_10_ copies/ million PBMC). Pre-therapy PBMC HIV-1 DNA levels did not differ significantly between ET (median, 3.3 log_10_ copies/ million PBMC, IQR: 2.8–3.6 log_10_ copies/ million PBMC) and LT (median, 3.7 log_10_ copies/ million PBMC, IQR: 3.4–3.9 log_10_ copies/ million PBMC) children. Pre-therapy PBMC HIV-1 log_10_ DNA levels correlated with pre-therapy plasma log_10_ HIV-1 RNA levels (r = 0.59, p<0.001), remaining statistically significant (p = 0.002) after adjustment for prior antiretroviral exposure and age at cART initiation using linear regression.

Over the first year of cART, PBMC HIV-1 DNA levels declined significantly (-0.91±0.08 log_10_ copies/ million PBMC, p<0.001) from pre-therapy levels. The estimated slopes for decline were -1.04±0.11 log_10_ DNA copies/ million PBMC in ET (p<0.001) and -0.74±0.13 log_10_ DNA copies/ million PBMC in LT (p<0.001); the slopes of decline did not differ significantly between ET and LT (p = 0.07) children.

**Fig 1:**
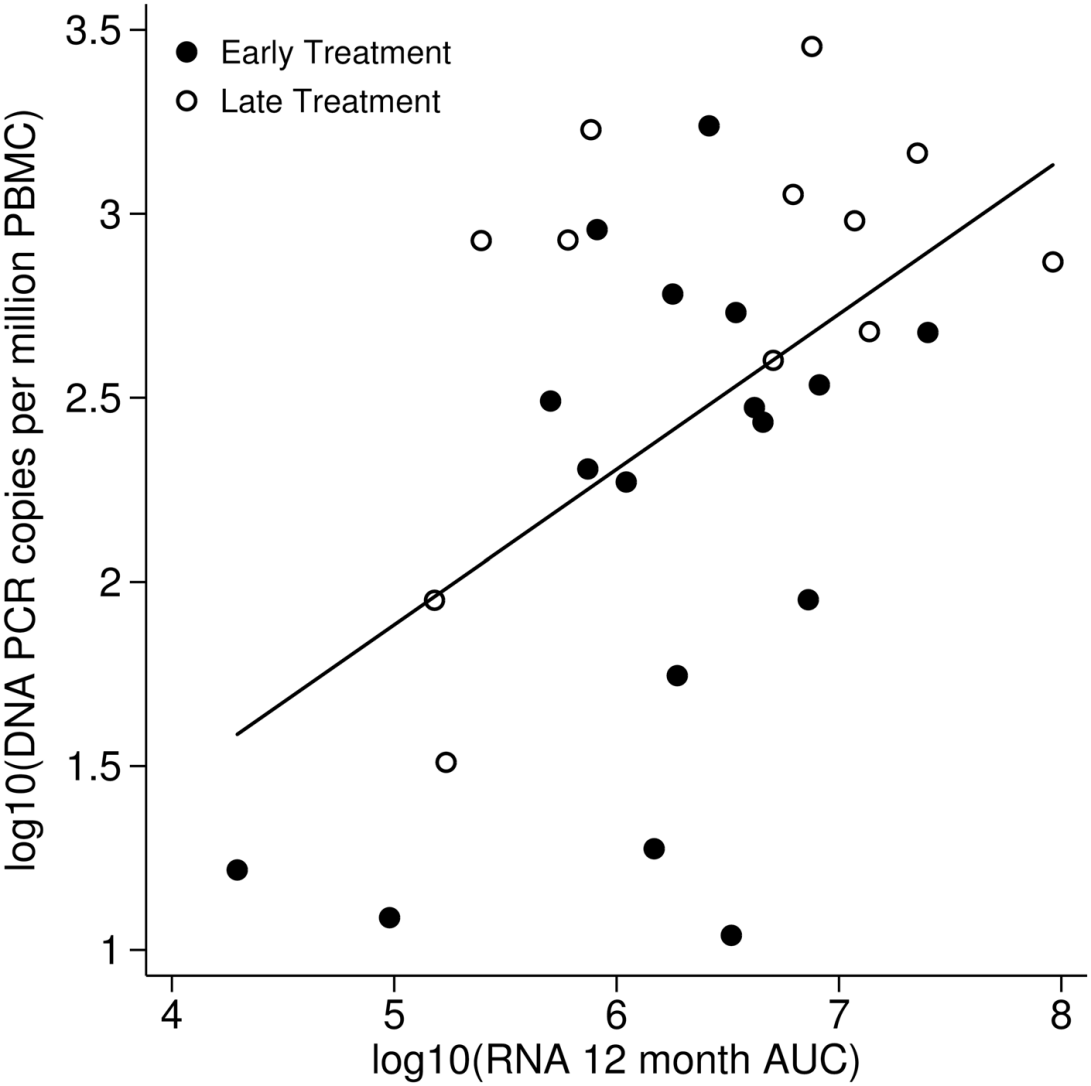
Exposure to HIV-1 replication and PBMC HIV-1 DNA levels one year following cART. The estimated 12 month AUC was significantly positively associated with HIV-1 DNA levels at one year (r = 0.51, p = 0.004).

Plasma HIV-1 RNA values were intensively monitored over the first year of cART and were used to calculate an area under the curve (AUC) as a more refined measure of exposure to viral replication. A median of 15 HIV-1 RNA values (range 11–18) were available through 12 months for each study subject and were included in the AUC calculations. Plasma HIV-1 RNA AUC log_10_ was significantly associated with PBMC HIV-1 log_10_ DNA levels at one year (r = 0.51, p = 0.004, Fig 1).

### Continued decline in PBMC HIV-1 DNA levels after one year of cART in virologic responders

Twenty-one children (13 ET, 8 LT) met the criteria for virologic suppression at one year and continued on cART (Table 1). There were no significant differences in pre-therapy plasma HIV-1 RNA levels, PBMC HIV-1 DNA levels, %CD4, and exposure to antiretroviral therapies between virologic responders and non-responders (data not shown). Pre-therapy plasma HIV-1 RNA levels in ET and LT responder children (median 5.3 in ET versus 4.8 log_10_ copies/ml in LT children; Table 1) and pre-therapy PBMC HIV-1 DNA levels (median 3.0 and 3.7 log_10_ copies/million PBMC, respectively; Table 1) did not differ significantly.

In 13 ET responder children, plasma HIV-1 RNA levels fell to <400 copies/ml at a median of 8 weeks after cART initiation (IQR: 8–12 weeks); median age at viral suppression was 4.2 months (IQR: 3.4–4.7 months). In 8 LT responder children, plasma HIV-1 RNA fell to <400 copies/ml at median 10 weeks after cART initiation (IQR: 3–12 weeks; not significant compared to ET); median age at viral suppression was 11.4 months (IQR: 8.4–15 months). In all 13 ET responder children, viral suppression was accompanied by loss of HIV-specific antibodies on enzyme-linked immunosorbent assay (ELISA) by 18 months of age; all 8 LT children remained HIV-1 seropositive by ELISA (data not shown).

PBMC HIV-1 DNA levels at years 1 and 4 were correlated with age at cART initiation (year 1: Spearman r = 0.45, p = 0.04; year 4: Spearman r = 0.57, p = 0.03) and age at which HIV-1 plasma HIV-1 RNA fell to <400 copies/ml (year 1: Spearman r = 0.49, p = 0.02; year 4: Spearman r = 0.61, p = 0.02) in responder children. In ET responder children, pre-therapy PBMC HIV-1 log_10_ DNA levels were significantly correlated with PBMC HIV-1 log_10_ DNA levels at year 1 (r = 0.87, p<0.001), 2 (r = 0.65, p = 0.02); and 4 years of cART (r = 0.85, p = 0.002); in LT responder children, pre-therapy PBMC HIV-1 log_10_ DNA levels were significantly correlated with PBMC HIV-1 log_10_ DNA levels at year 1 (r = 0.83, p = 0.01) and year 2 (r = 0.76, p = 0.03), but not at 4 years of cART (r = 0.86, p = 0.052).

**Fig 2:**
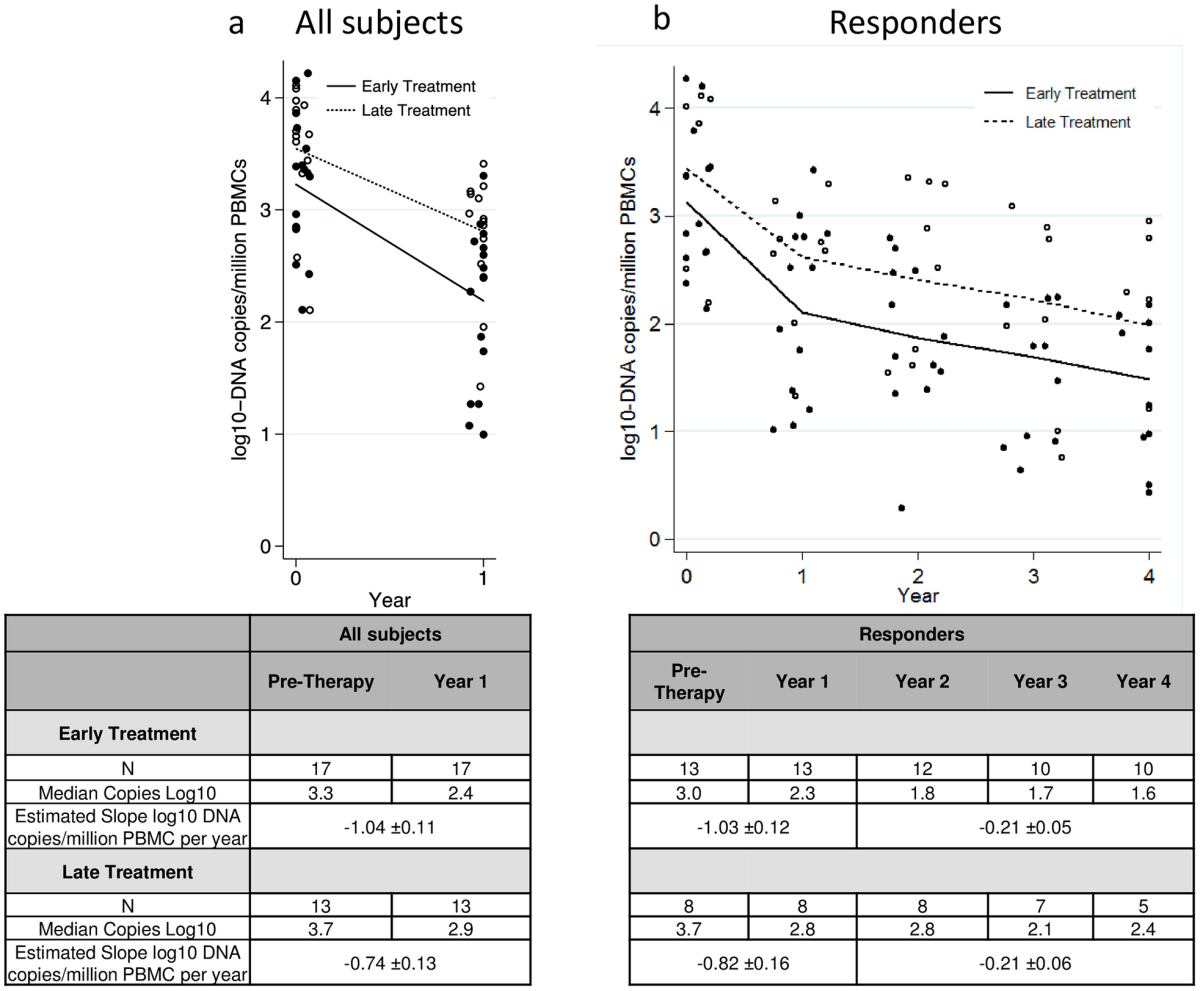
Estimation of PBMC HIV-1 DNA Decline. **a) Estimation of PBMC HIV-1 DNA Decline in All Children over the First Year of cART**. ET (closed circle and solid line) and LT children (open circle and dashed line) experienced a significant decline in PBMC HIV-1 DNA after one year of cART (-1.04±0.11 log_10_ DNA copies per million PBMC, p<0.001 in ET and -0.74 ±0.13 log_10_ DNA copies per million PBMC, p<0.001 in LT). **b) Estimation of PBMC HIV-1 DNA Decline in Children with Controlled HIV-1 Replication**. ET (closed circle and solid line) and LT children (open circle and dashed line) experienced a marked decline in PBMC HIV-1 DNA levels in the first year of cART (-1.03±0.12 log_10_, p<0.001 in ET and -0.82±0.16 log_10_, p<0.001 in LT), but the rates of decline did not differ significantly between ET and LT (p = 0.3). Similarly, in years 1 through 4 of cART, PBMC HIV-1 DNA levels continued to decline significantly in both the ET (p<0.001) and LT groups (p<0.001), but the decline slopes were not statistically significantly different between the groups.

Declines in PBMC HIV-1 DNA levels were observed in ET and LT responder children at 1 year of cART, with calculated slopes of -1.03±0.12 log_10_ DNA copies/million PBMC in ET (p<0.001) and 0.82±0.16 log_10_ DNA copies/million PBMC in LT (p<0.001); slopes of decline did not differ significantly between ET and LT (p = 0.3) and the absolute log_10_ DNA levels were not different in ET and LT responder children at 1 year of cART (p = 0.08). PBMC HIV-1 DNA levels continued to decline in years 1 through 4 in both the ET responder and LT responder children (slopes -0.21±0.05 log_10_ DNA copies/million PBMC per year in ET, p<0.001; -0.21±0.06 log_10_ DNA copies/million PBMC per year in LT p<0.001); again, the slopes of decline (p = 0.9) and year 4 PBMC HIV-1 DNA levels (p = 0.06) did not differ significantly between ET and LT children (Fig 2).

## Discussion

This study is among the first to quantify PBMC HIV-1 DNA levels in young children and to our knowledge, the first to precisely define the relationship between circulating levels of total PBMC HIV-1 DNA and duration of exposure to HIV-1 replication over the first year following cART initiation. Serial measurement of PBMC HIV-1 DNA levels in children with sustained virologic response allowed for the evaluation of continued decay of PBMC HIV-1 DNA levels up to 4 years following cART.

High levels of total PBMC HIV-1 DNA (median 3.4 log_10_ copies/million PBMC) were detected in all children prior to therapy. While our HIV-1 DNA quantification assay differed from those used in other recent studies, PBMC HIV-1 DNA levels measured in this study prior to therapy and at 1 year of cART were remarkably similar to those in 14 infants reported by Uprety et al using droplet digital PCR [16].

Pre-therapy HIV-1 DNA levels correlated with plasma HIV-1 RNA levels and a steep decline in PBMC HIV-1 DNA levels (median -0.91 log_10_ DNA copies/million PBMC) was observed over the first year of treatment. In children who achieved and maintained control of HIV-1 replication, the decline in PBMC HIV-1 DNA levels over the first year of cART (median slope -1.03 and -0.82 log_10_ DNA copies/million PBMC for ET and LT respectively) was greater than that measured after 1 year (median slope -0.21 and -0.21 log_10_ DNA copies/million PBMC for ET and LT respectively). The decline in PBMC HIV-1 DNA levels over the first year of cART were similar in magnitude to those reported over the first year of cART in adults treated within two months of infection [10, 17], and, more recently, in a small group of early-treated children [16]. These early changes in cell-associated HIV-1 DNA levels over the first year of cART likely reflect a reduction in new rounds of infection along with clearance of productively infected CD4+ T cells containing integrated HIV-1 DNA.

Continued measurement of PBMC HIV-1 DNA levels was conducted in a cohort of children (“responders”) with sustained plasma HIV-1 RNA < 50 copies/ml, suggesting near complete inhibition of HIV-1 replication. Moreover, all ET responder children seroreverted after 18 months, which has been shown to be a marker for effective early control of HIV-1 replication [2, 18] that correlates with low circulating HIV-1 DNA levels in older children [12]. Total PBMC HIV-1 DNA levels continued to decline through four years in both ET and LT responder children. This differs from the reported stability of PBMC HIV-1 DNA levels in children between one and two years of cART in a recent study [16]. Of note, 56% of infants in their study had intermittently detectable HIV-1 RNA in their plasma over the course of the study, suggesting ongoing viral replication or production from long-lived cells with integrated genomes.

Pre-therapy PBMC HIV-1 DNA levels did not differ significantly in ET and LT responder children. After four years of cART, total HIV-1 DNA levels trended lower in ET responders than in LT responders, but the study lacked sufficient power to detect differences between the groups. However, our finding that PBMC HIV-1 DNA levels at 1 and 4 years of cART were significantly correlated with age at cART initiation and age at virologic control is consistent with recent reports from two other studies of early therapy in infants [7, 16] and from a cross-sectional study in older children [12]. Measurement of HIV-1 RNA AUC over the first year of cART provided a more quantitative estimate of exposure to HIV-1 replication at early time points after cART initiation than previously reported, allowing us to discern a highly significant association between PBMC HIV-1 DNA levels at 1 year and plasma RNA AUC over the first year of treatment. Thus, our longitudinal studies more precisely define the relationship between exposure to HIV-1 replication in early primary infant infection and PBMC HIV-1 DNA levels after one year of treatment.

The number of participants included in the study (N = 30) is relatively small, but it is similar to the number of participants in recently reported studies [7, 16] and was sufficient for the original aims of demonstrating within subject treatment effects. The power to detect changes in HIV-1 DNA levels within participants exceeded 95% for both the entire group (N = 30) and the subgroup of responders (N = 21). The power to detect a difference in tests of association with plasma HIV-1 RNA was 82%, while the power to detect the differences in the rates of decline between ET and LT groups was reduced to 68% overall and 47% in responders. As noted, only limited data are available on HIV-1 DNA levels in children who have achieved viral suppression after early cART and who have available repository for longitudinal studies. These results will be useful for estimating effect sizes necessary for planning future studies characterizing the persistence of HIV-1 cellular reservoirs.

While cART reduced circulating PBMC HIV-1 DNA levels, HIV-1 DNA remained detectable in all children followed through 4 years of age. An important question is whether detectable HIV-1 DNA represents replication-competent virus capable of initiating HIV-1 replication in the absence of cART. PBMC HIV-1 DNA levels are estimated to be at least 100–150 times higher than levels of inducible provirus measured using viral outgrowth assays [16, 19]. While we did not directly measure the latent reservoir in this study, prior work has documented that early cART does not eliminate the latent reservoir [20] in infants. Moreover, the magnitude of reduction in latent reservoir size following early cART appears to be lower than the minimum 3–4 log_10_ reduction in reservoir size estimated in a recent model to predict periods of treatment-free remission [21]. Indeed, plasma HIV-1 RNA is readily detected within weeks of treatment discontinuation following early cART [22] and increases in circulating HIV-1 DNA levels have been reported following virologic rebound [7]. Thus, treatment discontinuation is not currently recommended for early-treated children outside of highly monitored research protocols.

Our data indicating a significant relationship between time to control of HIV-1 replication and residual HIV-1 reservoirs suggest that even earlier cART initiation may reduce long-lived HIV-1 reservoirs sufficiently to allow clinically significant periods of remission off treatment. The recently described case of the “Mississippi Baby” in which cART initiated within 30 hours of birth likely resulted in sufficient reduction of HIV-1 reservoirs to allow 27 months of remission off cART [23], provides additional support for this concept. Clinical trials of very early therapy (i.e., initiated within 48 hours of birth) are underway to evaluate whether this approach could provide a pathway for achieving long-term HIV-1 remission off antiretroviral therapy in children. These clinical trials will also provide an opportunity to further explore cell-associated HIV-1 DNA levels on cART as well as the relationship between measures of HIV-1 persistence and the potential for remission off therapy.
